# Complexity of diabetic nephropathy pathogenesis and design of investigations

**DOI:** 10.12861/jrip.2013.20

**Published:** 2013-06-01

**Authors:** Majid Tavafi

**Affiliations:** ^1^Department of Anatomy, Faculty of Medicine, Lorestan University of Medical sciences, Khoram Abad, Iran.

**Keywords:** Diabetic nephropathy, Pathogenesis, Treatment, Mediators

## Abstract

Diabetic nephropathy (DN) pathogenesis is very complex and multifactorial. There are several mechanisms or pathways that hyperglycemia leads to renal injuries. Each pathway makes renal injuries via several mediators. Some mediators are common between the pathways such as reactive oxygen species (ROS) and TGF-β and there are many overlaps and interference between the pathways. This review summarized complexity of DN pathogenesis and overlaps or interfering of mediators between the pathogenesis pathways. Besides, in the review suggested new designs of researches based on this complexity pathogenesis. The pathogenesis of DN is certainly very complex and multifactorial. From the overview of molecular mechanisms of DN pathogenesis, there are many pathways and many mediators with many interferences and overlaps between them. The focal point of this pathogenesis still unknown but it seems that RAAS system, oxidative stress and TGF-β relatively are common between these complex tangle webs of pathogenesis.

**Implication for health policy/practice/research/medical education:**

Diabetic nephropathy (DN) pathogenesis is very complex and multifactorial. There are several mechanisms or pathways that hyperglycemia leads to renal injuries. Each pathway makes renal injuries via several mediators. Some mediators are common between the pathways such as reactive oxygen species (ROS) and TGF-β and there are many overlaps and interference between the pathways. This review summarized complexity of DN pathogenesis and overlaps or interfering of mediators between the pathogenesis pathways.

## Introduction

Diabetic nephropathy (DN) is the common cause of end stage renal disease that characterized by the accumulation of extra cellular matrix in glomerular mesangium (glomerulosclerosis) and kidney interstitial tissue that eventually leads to renal failure (1).

Hyperglycemia triggers several mechanisms or pathways that explain briefly as follows:

1- Hemodynamic changes (increase of systemic and intraglomerular pressure). Because of activation of various vasoactive hormones such as renin angiotensin aldosterone system (RAAS) especially intra renal angiotensin II (Ag II) by mesangial cells (2), Transforming growth factor beta (TGF-β) activate and induce overproduction of mesangial matrix. Other vasoactive factors that activated in hyperglycemia include prostanoids, nitric oxide, vascular endothelial growth factor (VEGF) and endothelins (2,3).
2- Metabolic pathways.
a- Advanced glycation products (AGEs). In diabetic condition matrix proteins glycosylated nonenzymatically and change to irreversible product named AGEs. AGEs bind to AGEs receptors on mesangial cells and induce injury (4).
b- Activation of protein kinase C (PKC). In hyperglycemic conditions, PKCb forms and activate mesangial expansion via TGF-β, VEGF, ROS and AgII (5,6).
c- Polyol pathway. Glucose in mesangial cells change to sorbitol and sorbitol accumulation leads to NADPH depletion, decrease of NO, increase of PGE2, increase of AGEs, oxidative stress and activation of PKC (7).
3- Oxidative stress. Briefly, hyperglycemia leads to production of reactive oxygen species (ROS) especially superoxide anions. ROS induce renal injuries via cell membrane peroxidation, protein oxidation, renal vasoconstrictors, DNA damages, increase and activation of NF-кβ, activation of PKC, AGEs formation and TGF-β induction (8). Besides there are many agents that induce oxidative stress in mesangial cells that include AgII, TGF-β, Oxidized LDL, AGEs, Aldosterone, Amino acids and serotonin (9). For detail can read the review articles (10,11).
4- Activation of cytokines and growth factors such as VEGF, CTGF, TGF-β, IL-1, IL2, IL 18 and TNF-α (6,8).
5- Mesangiolysis/apoptosis. Mesangial cells respond differentially to a diabetic environment, some mesangial cells respond by increasing matrix production and other cells respond by undergoing apoptosis (9).
6- NADPH oxidase activation. High glucose concentrations increase the expression of the
NADPH oxidase that leads to renal oxidative stress and then promotes mesangial expansion by increasing the expression of fibronectin and collagen-1 in the kidney (12).
7- JAK/STAT pathway. (Janus kinase/signal transducers and activators of transcription).
Under high glucose conditions, JAK-2 and STAT-1, 3 and 5 activates and along with induces TGF-β and fibronectin synthesis in glomerular mesangial cells (13).
8-Role of adenosine (A) and adenosine receptors (AR). Diabetes mellitus induces A1-AR and A2a-AR mRNA expression and increase A1-AR and A2a-AR protein levels in the kidney (14). The activation of A2a-AR is renoprotective effects during diabetic conditions (15) and A2b-AR activation induce VEGF expression in kidney glomeruli (16). In addition, activation of A2b-AR mediates TGF-β1 release from the glomeruli of diabetic rats (17).
9**-**Peroxisome proliferator activated receptors (PPARs). PPARγ agonists have been noted to possess the therapeutic potential to prevent the development of DN by decreasing the TGF-β (18), by down regulating the expression of glomerular fibronectin and inhibiting ROS in glomeruli of diabetic mice (19) and by suppressing the expression of TGF-β, VEGF, PAI-1, type-IV collagen and ICAM-1 in the kidneys of diabetic rats (20).
10- Dopamine. The dopamine levels increased in the kidneys of diabetic mice, suggesting a possible role for dopamine in the pathogenesis of DN (21).

In order to inhibition of DN, many studies have been done based on the mentioned mechanisms. Some sample studies include; use of ACE inhibitor such as perindopril (22), ARB such as losartan (23) or combination of ACE inhibitor with ARB (24), inhibition of AGE receptors by aminoguanidine (25), inhibition of PKC-B by using ruboxistaurin (26), use of antibody TGF-B (27), inhibition of TNF-a by infliximab (28), use of NADPH oxidase inhibitor (29), use of A2A-AR agonist (15), inhibition of IGF-1 receptor by sandostatin (30), use of PPAR agonist (31) and use of different antioxidants (32,33).

Metabolic control of diabetes and control of hypertension with renin angiotensin blocker remain the most effective method to prevent the progression of glomerular problems of diabetes and to stop the progression of the early pathological features of DN. However, strict metabolic control and achieving euglycemia is often difficult, burden with complications and may be less effective at least for advanced stages of diabetic kidney (9).

Many studies have designed based on mechanisms pathogenesis, have established efficient when managed to experimental models of diabetes but the effectiveness and safety of these agents in human remains to be established.

In human, limit studies carried out by ACE inhibitors, ARBs (24), PKC-B inhibitor (30), TNF-α inhibitors (29), Anti TGF-β, AGE inhibitor (26) and antioxidants. From these studies, ACE inhibitor and ARBs showed the best results. Although antioxidants showed very beneficial effects in animals but in human did not show desirable effects (10).

**Figure 1:**
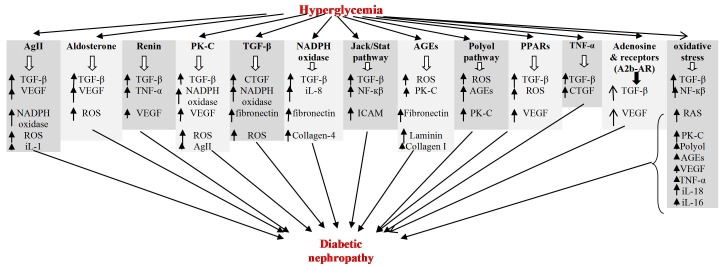
Tangle web of DN pathogenesis, note to pathways, mediators and interference or overlaps between them.

Mechanisms involved in diabetic nephropathy pathogenesis summarized in Figure 1 (6-10). Please see the Figure 1 carefully. You find that pathogenesis is more complex than our findings in the last. Briefly, about 13 major pathways showed in the Figure 1 but each pathway induces injury via several mediators or interaction with other pathways. Some mediators are common between major pathways such as TGF-β. Besides there are many overlaps between pathways and their mediators, for example AgII induce injury via oxidative stress and *vice versa* oxidative stress make injury by RAS, AGEs increase ROS and ROS increase AGES, NADPH oxidase increase TGF-β and *vice versa*, TGF-β increase ROS and ROS activate TGF-β. We do not know accurate mechanisms and contribution of each pathway in DN induction but human studies showed that inhibition of AgII is effective than inhibition of other pathways.

Annually new mediators detect in the mechanisms of DN pathogenesis and make pathogenesis more complex than the last. In the last researches, one or two of these pathways targeted but the desire results never have obtained. Because of many overlaps between pathways and mediators, total inhibition of DN via one or two agents seems impossible.

Theoretically, for total inhibition of all pathways at least needs to a super pill contains RAAS blocker, Antioxidant, TGF-β antibody and inhibitors of other pathways. However, use of the complex agents together may be having many adverse effects. The focal point of DN pathogenesis still is unclear and may be finding in future time. Do not forget that trigger of these pathways is hyperglycemia and the first step gold treatment must be blood glucose control via nutrient regime, sport, drugs, and insulin or plants blood lowering products.

Although good glycemic control may be the best prevention of DN, it develops in spite of treatment of diabetes (34). From the overview of molecular mechanisms of DN pathogenesis, it seems RAAS system, oxidative stress and TGF-β relatively are common between this complex tangle web of pathogenesis. Suggested to researchers that design new study on primates and then clinical trials by combination of multiple pathways inhibitors as a super pill and control of hyperglycemia or using multiproperty agent that inhinit some of the mechanisms pathogenesis.

## Conclusion

The pathogenesis of DN is certainly very complex and multifactorial. From the overview of molecular mechanisms of DN pathogenesis, there are many pathways and many mediators with many interferences and overlaps between them. The focal point of this pathogenesis still unknown but it seems that RAAS system, oxidative stress and TGF-β relatively are common between these complex tangle webs of pathogenesis. Recommend to researchers that choose agents that have multiproperty (blood lowering, RAAS inhibitor, antioxidant and …) or use super pills (collection of pathways inhibitors) in combat with DN because of complexity of DN pathogenesis. The blockade of one or two pathways or mediators cannot inhibit this complex pathogenesis.

## Author’s contribution

MF is the single author of the manuscript.

## Conflict of interests

The author declared no competing interests.

## Ethical considerations

Ethical issues (including plagiarism, data fabrication, double publication) have been completely observed by the author.

## Funding/Support

None.
